# Modified Valsalva test differentiates primary from secondary cough headache

**DOI:** 10.1186/1129-2377-14-31

**Published:** 2013-03-28

**Authors:** Russell JM Lane, Paul TG Davies

**Affiliations:** 1 Charing Cross Hospital, Imperial College, London, UK; 2 John Radcliffe Hospital, University of Oxford, Oxford, UK; 3 Department of Clinical Neurosciences, Charing Cross Hospital, Room 3 N12, Fulham Palace Road, London W6 8RF, UK

**Keywords:** Cough headache, Valsalva, Chiari malformation

## Abstract

**Background:**

The current definition of cough headache includes provocation of the symptom by Valsalva manoeuvre, and it is generally believed that all cough headache results from a sudden increase in intracranial pressure. We sought to question that presumption and to determine whether the Valsalva test might distinguish primary from secondary cough headache.

**Methods:**

We examined 16 consecutive cough headache patients using a modified Valsalva test (exhalation into the connecting tube of a standard anaeroid sphygmomanometer to 60 mm Hg for 10 seconds). A positive response was recorded if the manoeuvre provoked headache. All patients subsequently underwent brain MRI.

**Results:**

None of the patients had neurological signs. Eleven had positive modified Valsalva tests. Ten were found subsequently to have posterior fossa pathologies (*secondary* cough headache: 8 Chiari Type 1 malformations, 2 posterior fossa meningiomas). The cough headache was relieved following surgery in all cases. One patient with a positive Valsalva test had an apparently normal brain MRI but measurements of hindbrain and posterior fossa dimensions were consistent with ‘posterior fossa crowdedness’. The remaining 5 patients had negative (4 patients) or equivocal (1 patient) Valsalva tests and normal MRI scans (*primary* cough headache).

**Conclusions:**

These findings suggest that secondary cough headache results from a transient increase in intracranial CSF pressure during exertion in the presence of obstruction to normal cerebrospinal fluid dynamics. The modified Valsalva test can also determine whether tonsillar herniation found on brain MRI is symptomatic. Primary cough headache appears to be caused by a different mechanism, possibly through congestion of the orbital venous plexus in the presence of jugular venous incompetence and a reduced threshold for trigeminal sensory activation.

## Background

Most headaches are exacerbated by coughing, sneezing, straining and other exertions but some individuals experience headache *exclusively* with physical activities. Exertional cephalalgias comprise 1–2% of referrals to headache clinics [1,2] The current International Headache Classification (IHC2) [3] recognises three forms of exertion or activity provoked headache. The most common is ‘*primary exertional headache*’, defined under IHC2 as “a throbbing headache lasting from 5 minutes to 48 hours, occurring during physical exertion but not otherwise and not attributable to another disorder”. Such headache has sometimes been reported in relation to specific exertions and named accordingly (e.g. ‘weightlifter’s headache’). IHC2 considers *headache related to sexual activity* (coital cephalalgia) as a separate condition and moreover, distinguishes ‘pre-orgasmic headache’ from ‘orgasmic headache’, the latter having features of primary thunderclap headache. *Cough headache*, is defined under IHC2 as “headache of sudden onset, lasting from one second to 30 minutes, brought on by coughing, straining *and/or Valsalva manoeuvre*”. In addition, we have described previously, a number of cases of *exercise triggered migraine*, where headache fulfilling criteria for migraine, occasionally with aura, develops some time after exercise but not during the activity [4]. There is inevitably some degree of overlap between primary exertional headache and cough headache in terms of provocative activities. A recent review provides an excellent summary of these conditions and their treatment [5].

While historical and epidemiological evidence suggests that exercise-triggered migraine, exertional headache and coital cephalalgia are almost always benign, and probably manifestations of the migraine mechanism [1], cough headache is unique among headache disorders in that it is commonly associated with intracranial pathologies, most often posterior fossa abnormalities such as Chiari malformation [1]. The term *primary cough headache* (PCH) denotes patients where no relevant pathology is evident on brain imaging.

It is generally believed that all cough headache is caused by a sudden increase in intracranial pressure resulting from exertion [6]. While this seems likely for secondary cough headache cases, where structural pathology might alter CSF dynamics, the mechanism of PCH is unclear. We have examined the hypothesis that a Valsalva test might distinguish primary from secondary cough headache.

## Methods

We studied subjective responses to a modified Valsalva test in 16 consecutive patients with cough headache presenting to our general neurology and headache outpatient clinics over the last 5 years, and correlated the responses with subsequent brain MRI findings.

### Modified Valsalva test

The ‘standard’ Valsalva manoeuvre involves forced exhalation against a *partially* closed glottis to 40 mm Hg for 15 seconds and is widely used in the investigation of autonomic function and the management of supraventricular cardiac dysrhythmias [7,8]. Some variation in the method seems acceptable for pre-operative screening of autonomic function [9] but the standard procedure appears optimal in terms of efficacy in terminating supraventricular tachycardia [10].

During a Valsalva manoeuvre or a cough (i.e. paroxysmal expiration against a *closed* glottis), increased intra-thoracic and intra-abdominal pressure is transmitted to the large veins and thence rapidly to the paravertebral venous plexus, which dilates, compressing the spinal dura and increasing intrathecal pressure [11]. This pressure wave is transmitted cephalically, with a consequent increase in intracranial pressure. There is subsequently CSF flow in the reverse direction [11]. Normal subjects do not experience headache with coughing or other effortful activities but obstruction to this compensatory caudal flow caused by, for example, a Chiari malformation or posterior fossa tumour, can result in craniospinal pressure dissociation with intracranial dural stretching, resulting in headache [11].

When lumbar CSF pressures were measured pre-operatively in patients with Chiari malformations, with or without associated cough headache, the standard Valsalva manoeuvre did not increase CSF pressure more in the patients than in controls [12]. We reasoned therefore, that the standard manoeuvre was unlikely to be an adequate provocation for cough headache; coughing induces a particularly marked CSF pressure increase [11-13], with intra-abdominal pressures exceeding 100 cm H_2_O [14]. We therefore adopted a modified procedure, requiring greater exhalation force for a shorter period of time, as a closer approximation to the effort of coughing.

Each patient was asked to exhale into the spigot of the rubber connecting tube of an aneroid sphygmomanometer to a pressure of 60 mm Hg and to maintain this for 10 seconds. This approximates to a maximal voluntary Valsalva manoeuvre using such a device. Normal subjects experience mild light headedness and ‘head rush’ with this procedure but do not experience headache. However, some cough headache patients reported severe headache that closely resembled their primary symptom, resolving within a few minutes of stopping the forced exhalation. This was recorded as a ‘positive’ Valsalva test. If only non-specific symptoms occurred, as typically experienced by normal subjects, the test was considered negative. No patient suffered adverse effects from the procedure. Brain MRI was performed subsequently in all patients.

### Brain MRI

Standard brain MRI sequences, including sagittal images, were obtained in all patients and were interpreted by consultant neuroradiologists at our respective clinical neurosciences centres. Chiari 1 malformation is defined as ‘herniation of the cerebellar tonsils through the foramen magnum into the cervical spinal canal’ and this diagnosis was made without any specific restriction with regard to the extent of tonsillar herniation, since there is no consensus as to the minimum extent of herniation likely to produce symptoms (see Discussion).

## Results

Table 1 shows the results of Valsalva testing and MRI scan findings in the 16 patients, together with details of headache location, cough headache triggers, antecedent illness or events, and previous headache history. Individual case histories are provided in the Appendix.

**Table 1:** Response to modified Valsalva test and brain MRI results in 16 patients with ‘cough headache’

**No**	**Age**	**Sex**	**Location**	**Triggers**	**Immediate preceding history**	**Headache history**	**Valsalva test response**	**MRI brain**
1	22	F	Holocranial	Coughing, lifting, standing from lying	None	None. MO subsequently	+	Chiari 1
*2*	*28*	*M*	*R Frontal*	Coughing, laughing, sexual intercourse, bening over	*URTI*	*None*	±	*Normal*
3	*57*	*F*	*Holoccanial*	Coughing, sneezing, bending over, crouching	*URTI*	*Facial migraine, tension headache, auras*	-	*Normal*
4	30	F	Holocranial, R parietal	Standing up from a crouch	None	MO, exertional headache	+	Chiari 1
5	32	F	Occipital	Coughing, laughing, sneezing, bending over	None	MO	+	Chiari 1
6	65	M	Occipital	Coughing, bending over, stooping, lifting, straining at stool	None	Laughing headache	+	Chiari 1
*7*	*63*	*M*	*L Frontal*	Coughing, bending over	*URTI*	*MO*	-	*Normal*
*8*	*87*	*M*	*Frontal*	Coughing, sneezing	*None*	*None*	-	*Normal*
9	63	F	Vertex	Coughing, straining, laughing, bending over	None	MO	+	Posterior fossa meningioma
10	30	F	Occipitonuchal	Coughing	None	MO, thunderclap	+	Chiari 1
11	36	M	Frontal	Laughing, bending over, coughing	None	None	+	Chiari 1
12	43	F	Vertex	Coughing, sneezing, lifting	None	MO, MO with trigeminal autonomic symptoms	+	Chiari I
*13*	*38*	*M*	*L fronto-temporal*	Golf swing, bending over, lifting	*None*	*MA*	-	*Normal*
14	62	F	R frontal, any area	Coughing, bending over, sneezing, straining at stool	Cervical manipulation	None	+	‘Posterior fossa crowdedness’ ^1^
15	46	F	Occipital	Coughing, shouting, sneezing, bending over, turning quickly	None	None	+	Posterior fossa meningioma
16	32	F	Holocranial, frontal	Bending over, lifting, blowing up balloons	None	MO, exercise-triggered migraine	+	Chiari 1

10 patients had positive tests and abnormal MRI (8 Chiari 1 malformation, 2 posterior fossa meningiomas. Patient 14 had a positive test with normal MRI but had a high hindbrain to posterior cranial fossa ratio consistent with ‘posterior fossa crowdedness’ [14]. 4 patients had negative tests and one an equivocal response (primary cough headache, PCH). Patients 4, 13 and 16 had ‘cough headache’ but did not complain of headache with coughing.

Eleven of the 16 patients had a positive modified Valsalva test. Ten proved to have posterior fossa pathologies on MRI (8 Chiari 1 malformations, 2 meningiomas). None of the patients had neurological signs. Surgical treatment abolished the cough headache in all patients. The remaining patient (Patient 14) with a positive Valsalva test had an apparently normal brain MRI but measurements of the dimensions of the hindbrain relative to the posterior cranial fossa were consistent with ‘posterior fossa crowdedness’ [15]. Her cough headache resolved spontaneously after 6 months. Two of the patients with positive Valsalva tests and Chiari malformations (Patients 4 and 16), reported typical ‘cough headache’ provoked by a variety of effortful activities but had never experienced headache with spontaneous or voluntary coughing.

Four patients had negative Valsalva tests and normal MRI, consistent with PCH. One further patient (Patient 2) experienced mild head pain on Valsalva testing, not typical of the primary symptom, together with non-specific symptoms, but had a normal MRI. As with patients 4 and 16, Patient 13, with negative Valsalva test and normal MRI, had experienced headache with a variety of effortful activities but did not complain of headache with coughing.

The most common headache location reported in the secondary cough headache patients was occipital (4/11) while in the PCH patients it was frontal (4/5). However, headache location or other headache characteristics did not reliably distinguish the two groups. Three of the five PCH patients had developed cough headache during or following an episode of presumed upper respiratory tract infection, with severe, repeated coughing. The patient with ‘posterior fossa crowdedness’ had developed cough headache shortly after cervical manipulation for chronic neck pain. We cannot exclude the possibility that she might have suffered a vertebral dissection during the procedure but there was no evidence of this on MR cerebral angiogram at the time she was investigated for cough headache.

Patients 6 and 11 initially experienced headache provoked exclusively by laughing. Both proved to have Chiari malformations but Patient 6 had suffered ‘laughing headache’ as an isolated symptom some twenty years before the current presentation, which was characterised by headache provoked by a variety of activities, including coughing. Although the cough headache initially resolved following surgery he subsequently experienced a brief recrudescence of laughing headache.

## Discussion

In keeping with previous observations [1,15-17], we found that our primary and secondary cough headache patients could not be distinguished reliably by headache characteristics or location. While the headache was anterior in the majority of the PCH patients and most often occipital in the secondary cough headache patients, who all had posterior fossa pathologies, this was not consistent. Similarly, there were no consistent factors in the antecedent headache histories or immediate premorbid events, although periods of protracted coughing secondary to upper respiratory tract infections preceded the development of cough headache in three of our five PCH patients, an association noted previously by others [6].

By contrast, the modified Valsalva test was positive in all patients who proved subsequently to have structural pathology and was negative or equivocal in those with normal brain MRI.

All our patients experienced ‘cough headache’ with a variety of effortful provocations but paradoxically, some did not experience headache with coughing, despite a positive Valsalva test and culpable pathology. This illustrates the overlap between ‘primary exertional headache’ and ‘cough headache’ with respect to provocations, which in cough headache have been reported to include: coughing, nose blowing, breath holding, bending over [18], sneezing, straining at stool, laughing [19], lifting heavy objects and sexual activity [17]. Laughing was the sole provocation initially in two of our patients with Chiari malformation, a feature noted previously by others [20]. On this evidence, we question whether the term ‘cough headache’, introduced by Sir Charles Symonds in 1956 [19] is entirely appropriate for this entity and suggest that ‘effort headache’, the term first used in relation to this condition by Jules Tinel in 1932 [18], more closely reflects the diverse repertoire of provocations.

Although a number of cranial and intracranial pathologies have been associated with secondary cough headache [6], the commonest in our series was Chiari type 1 malformation. This is consistent with experience in a larger series reported by Pascual et al [1], although a study of similar size from Taiwan, undertaken at a similar time and of similar duration, found a far lower prevalence of secondary to primary cough headache cases, and an unusually low prevalence of Chiari malformations [2].

Headache is reported to occur in 28–70% of patients with Chiari 1 malformations [17,21]. Cough headache was found to be related to the extent of cerebellar tonsillar descent on MRI in one series [21] but not in another, where the association was with the peak CSF pressure generated by coughing [12]. Surgical decompression of the foramen magnum restores normal CSF dynamics in Chiari malformation and is reported to relieve secondary cough headache [12,21,22], as in the present series. We would emphasise that a detailed analysis of published evidence relating radiological and surgical anatomy to clinical symptoms in thousands of patients with Chiari malformation, shows that there is no minimal degree of tonsillar herniation that cannot be symptomatic while conversely, ‘radiologically signficant’ tonsillar descent may not be symptomatic [23]. In addition, Chiari 1 malformation is also associated with a greater than normal hindbrain to posterior cranial fossa volume ratio [15]. This is the defining characteristic of ‘posterior fossa crowdedness’, which can cause cough headache even in the absence of overt tonsillar herniation [15], as exemplified by Patient 14 in our series. We did not therefore impose any restriction with regard to the extent of tonsillar herniation for the radiological diagnosis of ‘Chiari malformation’.

It is commonly believed that the head pain in PCH is also caused by an increase in intracranial CSF pressure with exertion, as with secondary cough headache [5,6]. Lumbar puncture is reported to relieve PCH in some cases, consistent with this hypothesis [24]. However, this mechanism does not accord with our observation that PCH is not provoked by a maximal voluntary Valsalva manoeuvre. It could be argued that there is a ‘threshold’ for precipitating headache through increased intracranial CSF pressure and that this is not achieved in PCH by even a maximal voluntary Valsalva manoeuvre. However, seemingly minor exertions, such as simply bending over, can precipitate the headache in PCH, and ‘cough headache’ was provoked by a number of different exertions but not with coughing in several of our patients.

Alternatively, PCH might reflect *d’algie veineuse intracranienne* – pain arising from intracranial venous distension, as originally proposed by Tinel [18]. Gupta has suggested that PCH might be venogenic, generated by effort-induced choroidal venous congestion [25,26]. Primary cough headache is usually dramatic in onset, unlike secondary cough headache, where there may be a slight delay after exertion before the head pain starts [22]. The ocular choroid is drained by four or five vortex veins and coughing causes an instantaneous surge in ocular venous pressure. This does not normally cause discomfort because of autoregulatory control of venous tone in the orbital venous plexus [25], which is dependent on sympathetic fibres carried in the ciliary nerves. The long ciliary nerves are branches of the nasociliary nerve, which originates from the first division of the trigeminal and carries both somatic sensory and sympathetic fibers, and innervates the anterior cranium. The headache of PCH is most often frontally distributed and can be aborted by applying orbital pressure [25], as noted by Patient 7 in our study. It is conceivable that a protracted period of coughing due to respiratory tract infection, the use of angiotensin converting enzyme inhibitor drugs [1], or perhaps frequent episodes of primary headache, might lead to peripheral sensitisation, with reduced threshold for trigeminal activation. In keeping with this, trigeminal autonomic symptoms can occur during attacks of PCH [27,28]. The condition generally responds to indometacin [6]. While this therapeutic effect has been attributed to the drug’s ability to reduce CSF pressure, it has recently been shown that it also has a potent inhibitory effect on trigeminal sensory pathway activation [29,30].

However, the ‘venogenic’ hypothesis would also demand a degree of jugular venous incompetence. Knappertz [31] noted that functional valves are usually present in the jugular veins adjacent to the jugular subclavian junction and these prevent retrograde transmission of venous pressure to the head during effort. It is established however, that some individuals lack these valves [32] and Doepp et al reported that patients with undifferentiated exertional headache were significantly more likely to have jugular venous incompetence than controls [33].

## Conclusion

In conclusion, our data suggest that secondary cough headache is caused by a transient increase in intracranial CSF pressure due to obstruction of normal CSF dynamics. The symptom can be reproduced by a modified Valsalva test and is relieved by surgical correction of the culpable pathology. While no clinical evaluation could supplant the use of brain MRI in the investigation of exertional cephalagias, we have found the modified Valsalva test to be robust in the detection of significant posterior fossa pathologies and would recommend its use in clinical practice. In particular, if a patient is found to have Chiari malformation on brain MRI undertaken for the investigation of headache or some other problem, a negative modified Valsalva test would indicate that the malformation was not likely to be the cause.

By contrast, PCH is not provoked by Valsalva manoeuvre and must be due to another mechanism. This could be through orbital venous plexus congestion during effort in the presence of jugular venous incompetence and a reduced threshold for trigeminal sensory activation.

## Appendix

### Patient histories

### Patient 1

**Figure 1:**
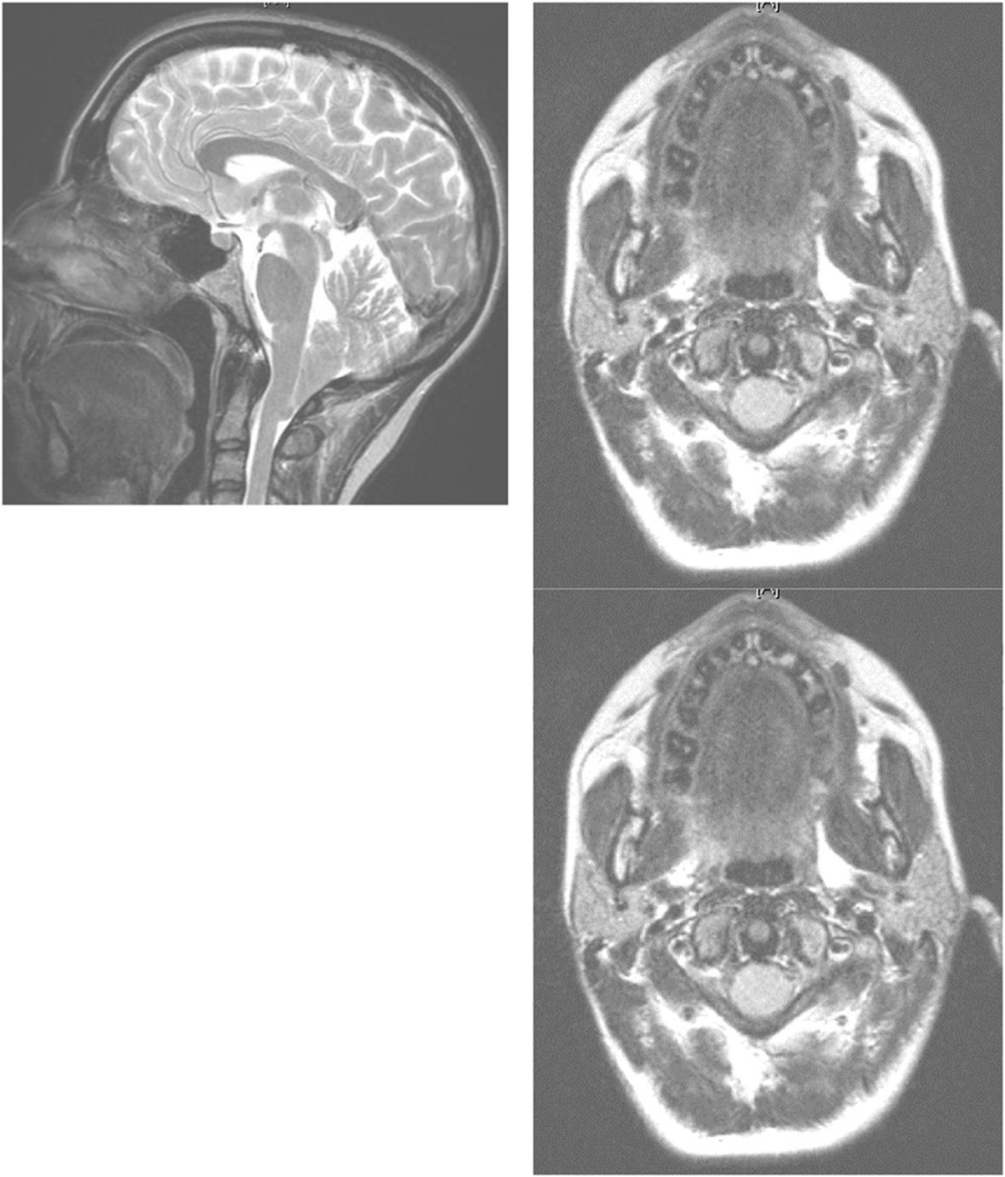
***Patient 1. ****Chiari I malformation causing secondary cough headache*. The cerebellar tonsils project 8 mm below the level of the foramen magnum (*left panel*) resulting in ‘crowding’ of structures at the cervicomedullary junction (*right panel*).

A 22 year old woman developed new onset persistent daily headache, throbbing in quality. It was located mainly in the occipital region and was notably worsened by head movements and by coughing and straining, for example when picking up her child. It was also precipitated when she stood up out of bed on waking. Her neurological examination was normal but she showed a strikingly positive Valsalva test, the manoeuvre precipitating an agonising attack of occipital headache that persisted for several minutes. MRI brain scan (Figure 1) showed a Chiari 1 malformation. She had no previous or family history of headache. The cough headache resolved completely following foramen magnum decompression and C1 laminectomy. However, 6 months later she presented following the onset of episodes of spontaneous headache typical of migraine without aura.

### **Patient 2**

A 28-year-old, gender reassignment genetically female patient presented with severe headache precipitated by coughing. He had suffered an upper respiratory tract infection three months earlier, causing repeated coughing before the onset of the cough headache. Coughing would induce a blindingly severe head pain in the right orbit and temporal area, graded 10/10, lasting about one minute, subsiding to 4/10 for up to two hours. There were no other symptoms associated with the headache. After some three weeks, he also began to experience milder headaches with a variety of other exertions, including laughing, sexual intercourse or simply on bending over. There was no previous or family history of headache. He had been taking testosterone for some years as part of his treatment programme but had not experienced headache as a result. Neurological examination was normal. Valsalva test produced minimal head pain, not typical of his primary complaint, and was graded ‘equivocal’. MRI brain scan was normal. He was lost to follow up.

### **Patient 3**

A 57-year-old woman was referred with headaches provoked by coughing, sneezing, straining at stool, bending over or crouching. These had begun following an upper respiratory tract infection, causing repeated coughing, six months previously. She described the headache as a ‘fullness’ or ‘dullness’ in the head, without specific localisation, which could take some minutes to dissipate. She had a background history of headaches, including episodes of facial migraine with mild trigeminal autonomic symptoms in her 20s, and weekly mild ‘tension-type’ headaches. She had also recently developed migrainous visual auras, usually without headache, although on one occasion an aura had been associated with headache precipitated by bending over. There was no family history of headache and examination was normal. Her Valsalva test was negative and MRI brain was normal.

### **Patient 4**

A 30-year-old woman presented with a two year history of a new headache problem on a background of typical but infrequent migraine without aura attacks dating from childhood. The new headache occurred only occasionally and was invariably provoked by standing up, having been stooped or crouched for a period of time. On other occasions, such postural changes would cause a ‘head rush’ sensation rather than pain. The attacks were *not* however, caused by coughing, straining or any other physical activity. The pain was described as excruciating, throbbing and located to the right parietal area, and was worsened by turning the head to the right. There was no visual obscuration. Once provoked, the headache would last for hours to days and was associated with nausea. Neurological examination was normal but she had a strongly positive Valsalva test that exactly reproduced the headache, except that the headache resolved much more quickly than the natural events. MRI brain scan revealed a Chiari 1 malformation but no cervical syrinx. There were no radiological signs of intracranial hypotension (meningeal enhancement, ‘brain sag’). She was offered foramen magnum decompression but declined and later emigrated.

### **Patient 5**

A 32-year-old woman presented with a two year history of headache provoked by coughing. The pain was occipital and would last a few seconds. For many months, coughing had been the only precipitant but for the year prior to presentation she had also had the headache following laughing, sneezing and bending over.

She had a background history of frequent and severe migraine without aura attacks and there was a strong family history of this. Neurological examination was normal but she had a positive Valsalva test, which reproduced the occipital head pain. MRI confirmed a Chiari 1 malformation, without syringomyelia. Her cough headache was completely relieved by sub-occipital craniectomy but her migraine attacks continued as before.

### **Patient 6**

A 65-year-old man presented with a 2-month history of throbbing headache in the occipital region, radiating to the temples, provoked by coughing, bending over, stooping, lifting heavy objects and straining at stool. Some 20 years earlier, he had consulted his GP complaining of frontal headache precipitated *exclusively* by laughing. He was not investigated at the time and the problem settled after a few years. There was no other personal or family history of headache. Neurological examination was normal but he had a strikingly positive Valsalva test, which reproduced his occipital headache. MRI brain scan revealed a Chiari 1 malformation, with tonsillar descent to the level of C2, without cervical syrinx. The occipital cough headache resolved following foramen magnum decompression and C1 laminectomy but during follow up, there was a mild recrudescence of the presumed primary cough headache precipitated by laughing and experienced anteriorly, although this stopped spontaneously after a few weeks.

### **Patient 7**

A 63-year-old man presented with a 6 month history of incapacitating paroxysmal headache every time he coughed. This had started following an upper respiratory tract infection, complicated subsequently by very frequent asthmatic coughing. Each coughing bout would be associated with very severe left frontal and periorbital pain, usually lasting less than a minute but sometimes more than 10 minutes, not associated with trigeminal autonomic symptoms. Occasionally, he would get a milder version of this headache when he bent down but he did not experience headache spontaneously, or when lifting, straining or with exercise.

He had suffered infrequent migraine without aura attacks some 20 years earlier but these had stopped completely when retired in his early 50s. Neurological examination was normal. Coughing voluntarily exactly reproduced the headache but the Valsalva test was consistently negative. Neuroimaging showed no relevant abnormalities. Intraocular pressures were normal. He had found that pressure over his left eyeball in anticipation of a cough seemed to prevent the headache, or speed recovery from the pain [see Discussion]. He was treated initially with a variety of interventions, including triptans, topiramate and beta blockers, to no benefit but the headache did seem to respond to indometacin. It eventually ceased completely after about 6 months.

### **Patient 8**

An 87 year old man, with no previous headache history, presented with a one year history of headaches precipitated by coughing or sneezing. The headache was frontal and very severe but would subside over about 15 minutes. He did not experience headaches under any other circumstance. There was no family history of headache. Examination was normal and Valsalva test did not reproduce the headache. Imaging was normal. Indometacin 25 mg three times daily prevented further headache attacks, which then stopped completely after 3 months.

### **Patient 9**

A 63 year old woman began to experience severe vertex headache precipitated by coughing, straining, laughing and bending over. The headache was maximal at the zenith of the exertion and declined rapidly afterwards. About 20 years earlier, she had experienced a period of frequent migraine without aura attacks but these had settled spontaneously. There was no other personal or family history of headache.

**Figure 2:**
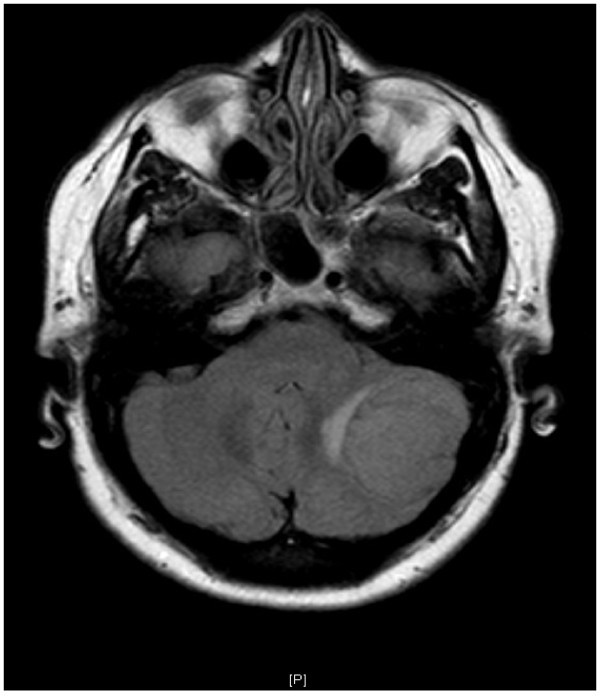
***Patient 9. ****Cough headache caused by left cerebellar hemisphere meningioma.* There were no abnormal signs apart from a strongly positive Valsalva test.

Neurological examination was normal but her Valsalva test was strongly positive. MRI brain scan revealed a left posterior fossa meningioma (Figure 2). The cough headache resolved completely after this was removed.

### **Patient 10**

A 30 year old woman complained that she had suffered recurrent headaches since her teens. These had become increasingly frequent and she had started to use analgesics regularly. In addition, she had begun to experience brief but severe headaches precipitated by coughing but not with other exertions. Two months before consultation, she had suffered a thunderclap headache. Investigations elsewhere, including CT head scan and CSF studies revealed no abnormality. When seen, she was asymptomatic and there were no abnormalities on examination. However, Valsalva test was strongly positive. MRI brain scan revealed a Chiari malformation without cervical syrinx. Her cough headache resolved following foramen magnum decompression and C1 laminectomy but she continued to experience chronic daily headache.

### **Patient 11**

**Figure 3:**
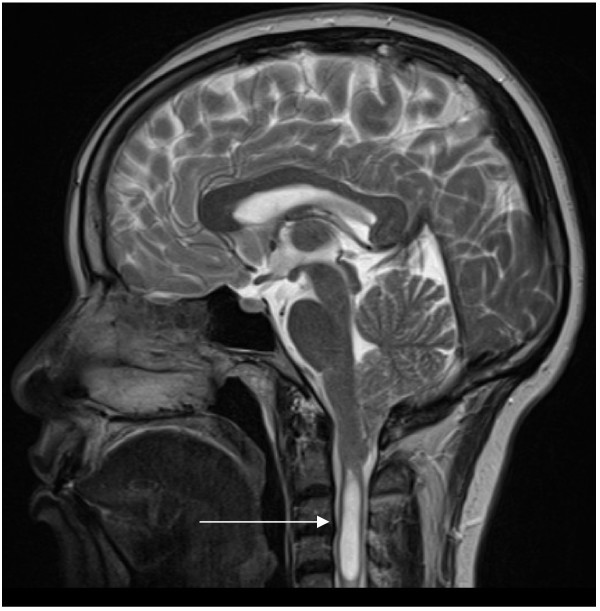
***Patient 11. ****T2 weighted saggital MRI brain and cervical cord showing marked Chiari 1 malformation and large cervical syrinx.*

A 36 year old woman gave a 2 year history of acute headaches precipitated exclusively by laughing, although not on every occasion, so that attacks would occur about twice a month. On such occasions, the bitemporal and frontal headache was of such severity that she would clutch her head and remain still until the pain receded, over about 5 minutes. There were no other symptoms. Seven months before presentation, she also began to experience this headache with bending over and coughing. There was no previous personal or family history of headache. Neurological examination was entirely normal but the headache was reproduced by the Valsalva test. MRI revealed a prominent Chiari I malformation and an extensive cervical cord syrinx (Figure 3). Her headaches resolved completely following foramen magnum decompression with C1 laminectory, and syringoperitoneal shunt insertion.

### **Patient 12**

A 43 year old woman was referred with a two year history of episodic intense, spontaneous orbital and periorbital headaches associated with ipsilateral eyelid drooping, conjunctival injection and epiphora, lasting up to two days. In addition, she gave a clear history of cough headache, brought on consistently by coughing, sneezing or lifting heavy objects for a similar period. However, this provoked headache was quite different from the spontaneous attacks, being located at the vertex and resolving within a few minutes of stopping exertion. She had suffered from typical migraine without aura since childhood. Valsalva test was strongly positive and MRI brain demonstrated Chiari I malformation. Foramen magnum decompression resulted in complete remission of the cough headache but she continued to be troubled by her primary headache attacks.

### **Patient 13**

A 38 year old man began to experience severe headache in the left front-temporal region associated with a variety of exertions, such as swinging a golf club and bending down whilst gardening, but generally only after a number of repetitions. However, he became concerned when the same headache was precipitated after lifting a heavy radiator on a single occasion. The headache was dull and non pulsatile, resolving after about 15 minutes. Between events he was otherwise asymptomatic. He had a previous history of typical migraine with visual aura and a strong family history of this condition. CT brain scan suggested possible Chiari malformation but Valslava test was negative. A subsequent MRI brain scan was entirely normal.

### **Patient 14**

A 62 year old woman, with no previous headache history, underwent cervical manipulation by a registered chiropractic for chronic neck pain caused by whiplash sustained in a road traffic accident 30 years earlier. She enjoyed a dramatic improvement but after 10 months, the discomfort returned and a second manipulation was undertaken. Three days later, she developed scalp formication with some mild background headache but in addition, very severe cough headache, provoked in addition by bending over, sneezing and straining at stool. The head pain was usually frontal but could occur elsewhere on occasions. Neurological examination was normal. The modified Valsalva test accurately reproduced the cough headache symptoms, which took two minutes to resolve.

MRI brain, MR angiogram and venogram were normal. However, skull and hindbrain measurements specified by Chen et al. [14] showed that this patient fulfilled the criteria for ‘posterior fossa crowdedness’ (Table 2). The patient was unable to tolerate indometacin. On follow up enquiry, she said that the cough headache had resolved spontaneously by 6 months

**Table 2:** Patient 14

	**Patient 14**	CH*	Controls*
PCF area (mm^2^)	3094	3164	3510
Hindbrain area (mm^2^)	2537	2463	2553
Clivus length (mm)	35	41.9	46.6
Clivus to mid pons (mm)	8	5.2	7.2
Basion to medulla (mm)	6.1	6.6	8.2

*Data from Chen et al. [14].

Measurements of posterior cranial fossa (PCF) area and linear dimensions relative to hindbrain area based on T1 weighted MRI, compared to published data from cough headache patients with apparently normal MRI (CH) and asymptomatic controls. Chen et al. [14] found that the PCF area in the cough headache patients was significantly smaller than controls while hindbrain areas did not differ. Thus the hindbrain/PCF ratio was significantly greater in the cough headache patients (0.78 ± 0.04 v 0.73 ± 0.06, p<005). In patient 14, the ratio was 0.82, well within the ‘posterior fossa crowdedness’ criterion.

### **Patient 15**

**Figure 4:**
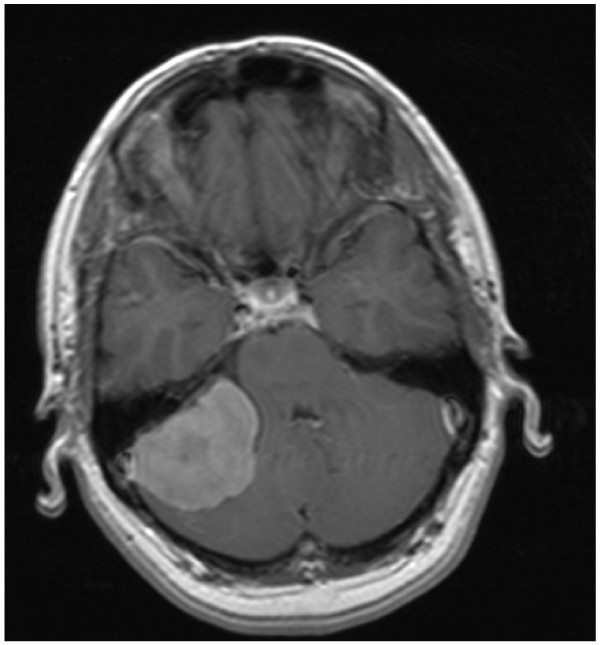
***Patient 15. ****Secondary cough headache. Meningioma at right petrous apex.* The patient was neurologically normal but had a positive modified Valsalva test.

A 46 year old woman presented with an 18 month history of severe occipital headache, lasting seconds, whenever she coughed, shouted, sneezed, bent down or turned around quickly, but not under any other circumstance. She had no previous history of headache or other notable illness. Neurological examination was entirely normal but the Valsalva test reproduced the headache. MRI brain scan revealed a large right petrous meningioma encroaching on the right internal acoustic meatus (Figure 4). The cough headache was relieved entirely by removal of the meningioma.

### **Patient 16**

A 32-year old woman presented with two headache syndromes, both dating from childhood: migraine without aura and headache associated with physical activities. The migraine with aura attacks had always been infrequent, occurring less than monthly but she would often experience such headaches some time after a sporting activity, such as netball, consistent with exercise-triggered migraine. In addition however, she would get very severe headache *during* a variety of exertions including bending over with arms extended, gardening, lifting heavy loads and especially, blowing up balloons. However, she denied ever experiencing headache with coughing. She had learned over the years, to avoid activities that might provoke headache. Both her sister and niece suffered from migraine.

She had a strongly positive Valsalva test and MRI brain demonstrated a Chiari 1 malformation without syrinx.

## Competing interests

The authors declare that they have no competing interests.

## Authors’ contributions

The patients in this study were ascertained by RL and PD through their general neurology and headache clinics. RL and PD undertook the clinical investigations described and requested the imaging studies. RL and PD both contributed to the data analysis and the writing of the manuscript, and all authors read and approved the final manuscript.
